# Improvement of microvascular complications in STZ-diabetic rats treated with *Pterocarpus erinaceus* Poir. extract

**DOI:** 10.1016/j.bbrep.2023.101541

**Published:** 2023-09-02

**Authors:** Kokou Atchou, Povi Lawson-Evi, Kwashie Eklu-Gadegbeku

**Affiliations:** Pathophysiology, Bioactive Substances and Safety Research Unit, Faculty of Sciences, University of Lome, Postbox 1515, Togo

**Keywords:** *Pterocarpus erinaceus*, Diabetes, Microvascular complications, Lipid profile, Renal fibrosis, Retinopathy

## Abstract

*Pterocarpus erinaceus Poir.* from Fabaceae family is a medicinal plant traditionally used in decoction or infusion to treat diabetes mellitus. Although this plant is used in treating diabetes, studies on the effectiveness of its stem bark on the complications induced by chronic hyperglycemia have not been thoroughly addressed. Thus, this study was conducted to prove the efficacy of hydroethanolic extract of stem bark of *P. erinaceus* on type 2 diabetes and its complications, such as renal fibrosis and retinopathy in rats. STZ diabetics. The dry extract of *P. erinaceus* stem bark was obtained following the hydroethanolic extraction (v/v). Diabetes was induced with streptozocin in SD rats pretreated with fructose-lard for 20 days. Then, the serum and urinary biochemical parameters were evaluated at the start and the end of the treatment. Rats with blood glucose ≥350 mg/dL and significant proteinuria were selected and treated with *P. erinaceus* stem bark extract and glibenclamide for 3 weeks. A complete blood count and a histopathological examination of the retina and kidneys were performed at the end of the 41st day of treatment. The results showed that *P. erinaceus* extract at a dose of 500 mg/kg bw and glibenclamide at a dose of 0.6 mg/kg bw caused a significant decrease (p < 0.0001) in basal blood glucose in STZ diabetic rats during treatment and improved oral glucose intolerance. At the end of the experiment, the treated rats showed a normalization in body weight, food and water consumption. Evaluating of biochemical parameters showed a significant (p < 0.001) decrease in total cholesterol, LDL-C, triglycerides, TG/HDL-C ratio, CPK and oxidative stress in treated rats. No retinal and kidney abnormalities were observed on histological sections in rats treated with plant extract and glibenclamide. In contrast, macular edema and renal fibrosis were observed in the diabetic control group. The findings showed that extract at a dose of 500 mg/kg bw improves oral glucose intolerance, and inhibits lipid deposition and retinal and renal fibrosis. Therefore, the plant extract could be exploited in the production of herbal medicines to manage diabetes and its complications.

## Highlights

• *In vivo* study of microvascular complications in a type 2 diabetes model for 41 days.
• Induction of metabolic syndrome and diabetic complications for 20 days in rats.
• Treatment of complications in STZ diabetic rats for 3 weeks with *P. erinaceus* Poir.
• Treatment improves insulin resistance and lipid profile.
• Treatment eventually reversed renal fibrosis and retinopathy.

## Graphical abstract

**Figure:**
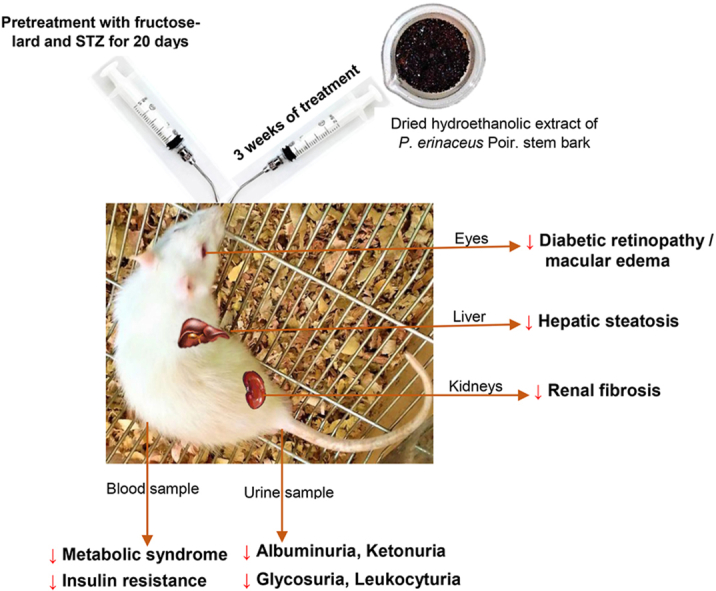

## Abbreviations

ADA: American Diabetes Association
AGEs: Advanced Glycation End-products
ALAT: Alanine aminotransferase
ANOVA: Analysis of variance
ASAT: Aspartate aminotransferase
AUC: Area under the curve
BRB: Blood-retinal barrier
Bw: Body weight
CKD: Chronic kidney disease
CPK: Creatinine phosphokinase
Cr: Creatinine
DNA: Deoxyribonucleic acid
EDTA: Ethylenediaminetetraacetic acid
ESRD: End-stage renal disease
GFR: Glomerular filtration rate
GLUT: Glucose transporter
GSH: Glutathione
H&E: Hematoxylin and eosin
HCT: Hematocrit
HDL-C: High-density lipoprotein cholesterol
HGB: Hemoglobin
HTC: Hematocrit
IDF: International Diabetes Federation
ip: Intraperitoneal
LDL-C: Low-density lipoprotein cholesterol
MCH: Mean corpuscular hemoglobin
MCHC: Mean corpuscular hemoglobin concentration
MCV: Mean corpuscular volume
MDA: Malondialdehyde
OGTT: Oral glucose tolerance test
PLT: Platelets
RBC: Red blood cells
ROS: Reactive oxygen species
SD: Sprague Dawley
STZ: Streptozocin
TG: Triglycerides
Total-C: Total cholesterol
VEGF-PEDF: Pigment epithelium-derived factor
WBC: White blood cells

## 1 Introduction

Diabetes complications occur after several times in case of poorly controlled blood glucose and with the age of the disease. Whether the diabetes is related to insulin secretion deficiency or insulin resistance, chronic hyperglycemia with fasting blood glucose >7.0 mM/L or postprandial blood glucose >11.1 mM/L damages multiple organs and leads to macro- and micro-vascular complications [1,2]. Type 2 diabetes, characterized by insulin resistance, is the most common and occurs in 90–95% of cases out of a total prevalence of 10.5% of the disease [1]. It leads to chronic complications that evolve silently and can cause premature death linked to cardiovascular risk in 50–80% of cases [[3], [4], [5]]. Indeed, hyperglycemia stimulates a cascade of multiple mechanisms and complex pathways that induce oxidative stress and toxic glycosylation products that affect neurons and impair blood vessel viability [6]. Microvascular dysfunction is most common in type 2 diabetes and leads to nephropathy, retinopathy, neuropathy, sensory loss, and leg amputation [7,8]. Among these complications, nephropathy and retinopathy respectively occupy proportions of 40% and 35.4% [9,10]. These two pathologies modify the lifestyle of patients, lead to increased health costs and render them disabled by the onset of blindness and end-stage renal disease (ESRD) associated with high morbidity and mortality [[10], [11], [12]]. Despite several types of therapies for diabetes, there is currently no specific drug to reverse or manage the progression of complications induced by hyperglycemia. Medicinal plants are a source of bioactive compounds and can be explored for this purpose.

*Pterocarpus erinaceus* Poir. is a medicinal plant used in traditional medicine to treat several types of diseases including diabetes [13,14]. Several parts of the plant are used. The stem barks are traditionally used in the treatment of ulcers, rheumatism, dermatitis and infections. The leaves are used against neurological disorders, fever, headache, malaria, abdominal pain, sexual infections and as an aphrodisiac to combat impotence. The roots are used mainly in the treatment of stomach ache, anemia, constipation, hemorrhoids and painful menstruation [14,15]. The stem bark of the plant is used as a decoction or infusion by the people of the maritime region of Togo to treat diabetes. Previous studies have reported the hypoglycemic and antioxidant activities of the plant, but very little data relates to its effect on diabetes-related complications [13,16]. Thus, we hypothesized that *P. erinaceus* inhibits diabetes complications, and we aimed to study the effect of hydroethanolic extract of the stem bark of the plant on diabetes and its two complications which are renal fibrosis and retinopathy.

## 2 Materials and methods

### 2.1 Chemical and reagents

All the solvents used were obtained from the commercial agency of ProLabo Diagnostics in Lome (Togo). The biochemical reagents were Cypress diagnostics and were used on the CYAN SMART CY009 Spectrophotometer, Germany. Hematology reagents and Auto Hematology Analyzer (URIT-5160) were from URIT Medical Electronic CO., LTD, India. The extract of *P. erinaceus* was prepared locally.

### 2.2 Animals

Male and female Sprague Dawley (SD) rats (200 ± 10 g) were used for the pharmacological studies. They were provided by the animal facility of the Department of Animal Physiology, University of Lome. The animals were sex-randomized in cages and then acclimatized to a temperature of 25 ± 2 °C with a relative humidity of ∼50% under a 12/12 h light/dark cycle for two weeks with access to food and water *ad libitum*.

### 2.3 Ethnobotanical survey and identification of the plant

A semi-structured ethnobotanical survey was conducted among 50 herbalists in the markets of the maritime region of Togo to identify the plants sold for the treatment of diabetes. Most of these were in Lome markets. During this survey, several plants were listed, but with gradual elimination, *Pterocarpus erinaceus* was selected for this study. The most widely used drug is the stem bark of *P. erinaceus*. Herbalists sell the dry bark of the stem of the plant which they offer patients to use as a decoction or infusion with an initial dose of 50 mL, twice a day. They recommend that patients check their blood sugar at least 1 or 2 times a week to gradually adjust the doses.

### 2.4 Collection of plant samples and identification

The fresh barks of the stem of *Pterocarpus erinaceus* Poir. were harvested in the AVE prefecture of Togo and identified at the University of Lome in the Laboratory of Botany and Plant Ecology. The voucher specimen has been deposited in the herbarium of this Laboratory under the number 15515 TOGO. The name has been checked with http://www.worldfloraonline.org, on July 7, 2023, and its status is *Pterocarpus erinaceus* Poir. of the Fabaceae family.

### 2.5 Extract preparation

The stem barks of *P. erinaceus* were pruned and rinsed with running water before being dried at a temperature of 20 ± 2 °C in the safe of the light for two weeks. The dried barks were reduced to powder and macerated in a hydroethanolic solution (50% ethanol; 50% water) for 72 h. The macerate was filtered on cotton, then on Whatman filter paper (Ø 150 mm) before evaporating to dryness at a temperature of 45 °C under a vacuum using a rotary evaporator (Buchi™, Germany). The dry hydroethanolic extract was recovered with a yield of 14.21% and stored at a temperature of 4 ± 2 °C in non-transparent glass bottles for the tests.

### 2.6 Experimental design

Diabetes and complications were studied in male and female SD rats for 41 days, subdivided into two phases: a first phase which corresponded to the induction of diabetes and complications over 20 days (D0–D19) and a second phase which corresponded to the treatment of diabetic rats with the plant extract and the reference drug which is glibenclamide for 21 days (D20–D40).

### 2.7 Induction of metabolic syndrome, diabetes and complications

Fructose-lard (6 g: 50 mL) was administered to the rats of both sexes at a rate of 20 mL/kg/day, divided into two doses for 20 days according to the method of Kadebe et al. [17], followed by modifications; to cause metabolic syndrome. On the 12th day (D11), streptozocin (STZ) at 50 mg/kg bw was injected intraperitoneally (ip) into the rats under fructose-lard to induce diabetes. The normal control (NC) group received distilled water orally and saline ip. The glycemia was measured on days D0, D11, D14 and D19 in all the rats fasted for 14 h.

### 2.8 Treatment and testing

Urine and blood samples were collected for biochemical and hematological parameters at the end of the 19th day which marked the start of treatment and on the 41st day of the end of the experiment. Urine was collected directly from the metabolic cage of the rats. Blood was collected from retro-orbital sinuses in rats using capillary tubes in dry tubes and EDTA tubes for biochemical and hematological analyses. At the end of induction of diabetes complications (D19), the urinary glucose, ketones, proteins, blood, leukocytes, pH and density were sought by qualitative (semi-quantitative) tests and the serum urea, creatinine, AST, ALAT, CPK, triglycerides, total cholesterol, HDL-cholesterol and LDL-cholesterol were determined using a spectrophotometer. Only rats of both sexes with blood glucose ≥350 mg/dL, significant proteinuria and other complications (nephrotic and cardiovascular risks and metabolic acidosis) were selected for treatment. The male and female rats were divided into equal numbers in the groups of 6 rats and then treated orally with the extract at a dose of 500 mg/kg bw and glibenclamide 0.6 mg/kg bw for 3 weeks (D20–D40). The previous NC has been renewed in this second phase which concerns the treatment. The 500 mg/kg bw extract of *P. erinaceus* was chosen as the active dose based on a previous study on oral glucose tolerance in mice [13]. Blood glucose was measured at the end of each week (D27, D34, D41) in rats fasted for 14 h for 3 weeks from the caudal vein using an Accu check active glucometer, Germany. At the end of the treatment, rats were fasted for 14 h, the urine was collected for qualitative tests, and an oral glucose tolerance test (OGTT) was performed by overloading with 2 mg/kg of glucose. After OGTT, rats were anesthetized with sodium pentobarbital and blood was collected into dry and EDTA tubes. Then, rats were sacrificed by decapitation, and an autopsy was performed to remove the liver, heart, lungs, brain, kidneys and eyes. Their weights were determined, then the liver, one kidney and one eye were frozen to determine oxidative stress markers. The remaining kidney and eye were fixed in 10% formalin for pathological histology studies.

Blood collected in EDTA tubes was used for a complete blood count on Auto Hematology Analyzer (URIT-5160). Then, the blood collected in dry tubes was centrifuged and the sera were used for the assay of the biochemical parameters using CYAN SMART CY009 Spectrophotometer. The following parameters were assayed according to the methods indicated on the Cypress diagnostic reagent data sheets: Urea (Urease GLDH – UV), Creatinine (Jaffe), AST (Aspartate/α-ketoglutarate IFCC), ALT (Alanine/α-ketoglutarate IFCC), CPK (Creatine-phosphate/activated NAC), Triglycerides (GPO-POD), Total Cholesterol (CHOD-POD); HDL-C (Direct).

Concerning oxidative stress markers, total proteins, MDA and GSH were assayed in liver, kidney and eye homogenates. Total proteins were assayed by the colorimetric method based on binding Coomassie brilliant blue to the protein and measuring the absorbance of the resulting complex at 595 nm [18]. MDA was assayed by reaction with thiobarbituric acid (TBA) and measurement of the absorbance of the resulting color at 586 nm [19]. GSH was assayed according to Ellman's method. The reaction with the SH groups leads to the reduction of 5,5′-dithiobis-(2,-nitrobenzoic) acid. The formation of nitromercaptobenzoic acid during the reaction causes a yellow color to appear which can be measured at 412 nm [20].

The histopathological examination was performed on the kidney and eye sections. The organs were removed from formalin and then embedded in paraffin. The blocks were cut into 5 μm thick sections and then spread on microscope slides. Then, the preparations were stained with hematoxylin and eosin (H&E) and observed under a light microscope at 400 magnification [21].

### 2.9 Statistical analysis

GraphPad Prism 6 (Software Inc., USA) was used to perform and analyze the data. Differences between groups were analyzed using one-way and two-way ANOVA and considered significant at p < 0.05. Values were expressed as mean ± SEM.

## 3 Results

### 3.1 Antidiabetic activities

**Fig. 1:**
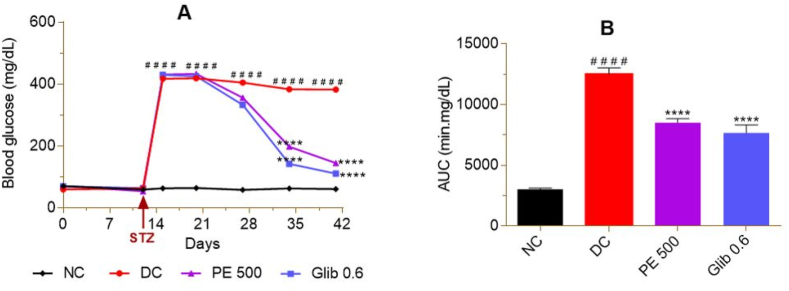
Effect of *P. erinaceus* extract on basal blood glucose in rats. A = blood glucose versus time; B = area under the curve of blood glucose. NC = normal control; DC = diabetic control; PE 500 = treated with *P. erinaceus* extract 500 mg/kg bw; Glib 0.6 = treated with glibenclamide 0.6 mg/kg bw. The blood glucose was measured in the fasting rats for 14 h on days D0, D12, D15, D120, D27, D34, D41. Values were analyzed with 2-way ANOVA and then presented as mean ± SEM. ^# # # #^p < 0.0001 vs. NC; ****p < 0.0001 vs. DC. n = 6.

Oral administration of fructose-lard at a dose of 20 mL/kg/day for 20 days (D0–D19) did not cause a significant increase in basal blood glucose in rats compared to normal controls (NC). On the other hand, the injection of streptozocin (STZ) in rats on D11, induced 3 days later (D14), a significant increase (p < 0.0001) in basal blood glucose (∼563.23%). This increase in blood glucose was maintained almost constant until the end of the 41 days of experimentation in untreated diabetic controls (DC). In diabetic rats treated with *P. erinaceus* extract for 3 weeks (D20–D40), there was a significant decrease (p < 0.0001) in blood glucose (D34 = 48.35%; D41 = 61.17%) compared to DC. The reference drug, glibenclamide, also caused a significant (p < 0.0001) decrease in basal blood glucose during treatment (D34 = 62.88%; D41 = 70.98%) (Fig. 1A). The area under the curve (AUC), which measures the availability of blood glucose per unit time, confirmed this significant (p < 0.0001) decrease in basal blood glucose levels in the treated groups compared to the DC (PE 500 = 31.68%; Glib 0.6 = 38.48%) (Fig. 1B).

**Fig. 2:**
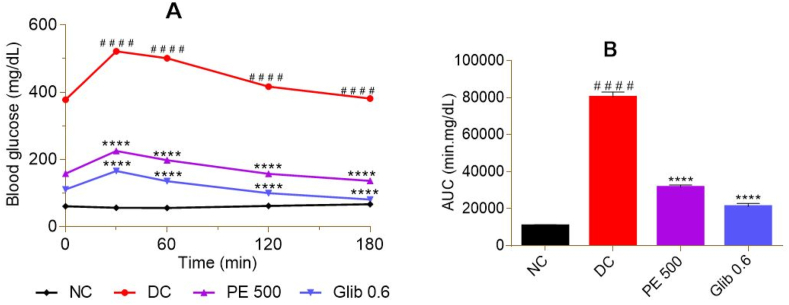
Effect of *P. erinaceus* extract on glucose intolerance in rats. A = blood glucose versus time; B = Area under the curve (AUC) of blood glucose. NC = normal control; DC = diabetic control; PE 500 = treated with *P. erinaceus* extract 500 mg/kg bw; Glib 0.6 = treated with glibenclamide 0.6 mg/kg bw. On the 41st day after the end of the treatment, the rats were subjected to the oral glucose tolerance test and received 2 g/kg bw of glucose per os after 14 h of fasting. Blood glucose was measured over 180 min t0 = 0 min corresponds to the rat basal blood glucose; t30, t60, t120, and t180 correspond to the measurement of blood glucose at 30, 60, 120 and 180 min after oral glucose overload. Values were analyzed with 2-way ANOVA and then presented as mean ± SEM. ^# # # #^p < 0.0001 vs. NC; ****p < 0.0001 vs. DC. n = 6.

The administration of 2 g/kg bw of glucose during the oral glucose tolerance test (OGTT) led, 30 min later, to a significant increase in blood glucose before gradually decreasing in treated and untreated rats (Fig. 2A). Compared to NC, this increase in blood glucose was significant (p < 0.0001) in DC over the 180 min (Fig. 2A). The groups that received extract and glibenclamide, 30 min before OGTT, showed a significant (p < 0.0001) decrease in blood glucose over the 180 min compared to DC (Fig. 2A). In these treated groups, the decrease in blood glucose was below their initial value at time t = 180 min (PE 500: 13.65%; Glib 0.6: 27.22%). The AUC confirmed this significant reduction (p < 0.0001) in glucose intolerance in the treated groups compared to the DC (PE 500: 60.74%; Glib 0.6: 73, 72%) (Fig. 2B).

### 3.2 Diabetes symptoms

**Fig. 3:**
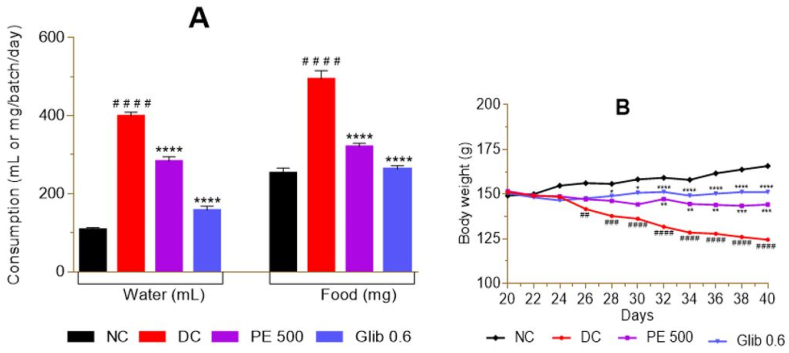
Effect of *P. erinaceus* on diabetes symptoms. A = water and food consumption; B = body weight change. NC = normal control; DC = diabetic control; PE 500 = treated with *P. erinaceus* extract 500 mg/kg bw; Glib 0.6 = treated with glibenclamide 0.6 mg/kg bw. The three parameters were measured in rats daily for 21 days during the treatment. Values were analyzed with 2-way ANOVA and then presented as mean ± SEM. ^# #^p < 0.01, ^# # #^p < 0.001, ^# # # #^p < 0.0001 vs. NC; *p < 0.05, **p < 0.01, ***p < 0.001, ****p < 0.0001 vs. DC. n = 6.

During the last 3 weeks of the experiment, there was a significant (p < 0.0001) increase in water and food (Fig. 3A) consumption, followed by a gradual decrease in body weight (Fig. 3B) in untreated diabetics rats (DC) compared to NC. When compared to DC, there was a significant (p < 0.0001) decrease in water and food consumption, with progressive body weight gain in rats treated with *P. erinaceus* extract and glibenclamide (Fig. 3A and B).

### 3.3 Metabolic syndromes

**Fig. 4:**
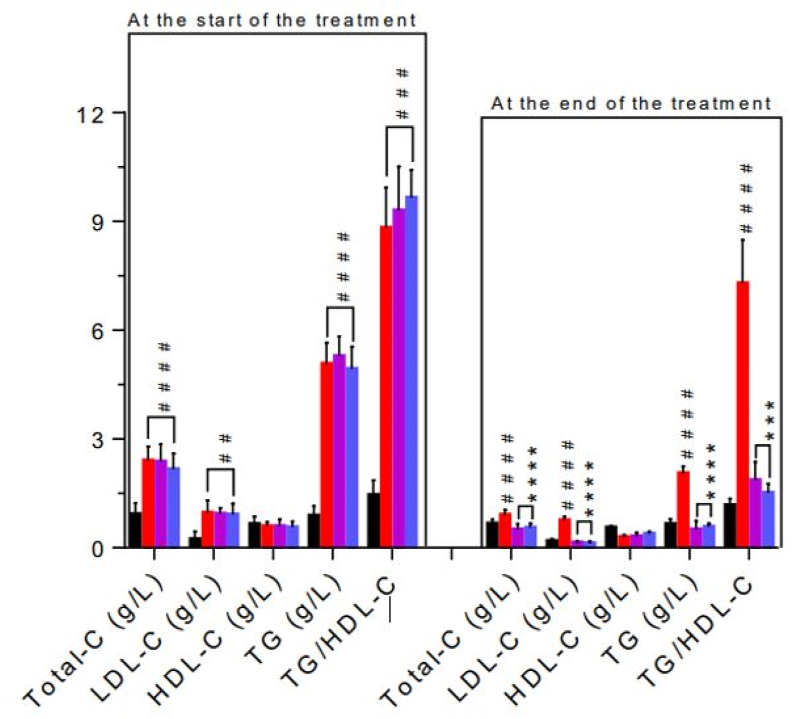
Effect of *P. erinaceus* on the lipid profile at the start and end of treatment. NC = normal control; DC = diabetic control; PE 500 = treated with *P. erinaceus* extract 500 mg/kg bw; Glib 0.6 = treated with glibenclamide 0.6 mg/kg bw. TG = triglycerides; Total-C = total cholesterol; HDL and LDL-C = high and low-density lipoprotein cholesterol. Serum biochemical parameters were assessed at the treatment's start (D20) and end (D41). Values were analyzed with 1-way ANOVA and then presented as mean ± SEM. ^#^p < 0.05, ^# #^p < 0.01 vs. NC; *p < 0.05, **p < 0.01 vs. DC. n = 6.

The lipid profile was very disturbed at the start of treatment in all diabetic rats. Levels of triglycerides, total cholesterol and LDL-cholesterol were significantly (p < 0.0001) elevated at the start of treatment in all groups (DC, PE 500, Glib 0.6) compared to the normal control group (NC) (Fig. 4). The TG/HDL-C ratio, which predicts the metabolic syndrome, was also significantly (p < 0.001) elevated at the start of treatment in diabetic rats fed fructose-lard. After 21 days (3 weeks) of treatment, these parameters decreased in all groups; but they remained significant (p < 0.0001) in the diabetic control (DC) when compared to the NC (Fig. 4). The groups treated with the extract and glibenclamide showed a significant decrease (p < 0.0001) in triglycerides, total cholesterol and LDL-cholesterol compared to DC (Fig. 4). Although there was no significant decrease in HDL-C, the TG/HDL-C ratio showed a significant (p < 0.001) reduction in the groups treated with the plant extract and glibenclamide (Fig. 4).

### 3.4 Diabetes complications

The urinary biochemical parameters were sought at the start and the end o the treatment. Before treatment, the urine of all diabetic rats showed the presence of glucose, ketones, proteins, blood, leukocytes and a decrease in pH. These parameters disappeared in the groups treated with the extract and glibenclamide (PE 500 and Glib 0.6). Only the diabetic control (DC) still presented these urinary parameters at the end of treatment (Table 1).

**Table 1:** Urinary biochemical parameters.

Parameters	NC	DC	PE 500	Glib 0.6
**At the start of treatment**				
**Glucose (mM)**	–	**45.833 ± 14.743**	**50.833 ± 13.189**	**55.333 ± 12.374**
**Ketones (mg/dL)**	–	**30.000 ± 3.162**	**27.500 ± 1.118**	**42.500 ± 2.500**
**Protein (mg/dL)**	–	**53.333 ± 14.757**	**51.677 ± 15.366**	**55.000 ± 14,318**
**Blood (/μL)**	–	**48.333 ± 3.801**	**63.333 ± 8.819**	**43.333 ± 6.146**
**Leukocytes (/μL)**	–	**70.883 ± 12.001**	**72.500 ± 12.366**	**74.167 ± 11.285**
pH	7.333 ± 0.211	**6.667 ± 0.167**	**6.583 ± 0.154**	**6.667 ± 0.167**
**Density**	1.007 ± 0.003	1.055 ± 0.030	1.012 ± 0.004	1.009 ± 0.003
**At the end of the treatment**				
**Glucose (mM)**	–	**49.600 ± 5.400**	**-**	**-**
**Ketones (mg/dL)**	–	**23.000 ± 5.148**	**-**	**-**
**Protein (mg/dL)**	–	**38.000 ± 15.937**	**-**	**-**
**Blood (/μL)**	–	**36.000 ± 11.000**	**-**	**-**
**Leukocytes (/μL)**	–	**71.000 ± 16.613**	**-**	**-**
pH	7.417 ± 0.154	**6.500 ± 0.224**	**7.250 ± 0.250**	**7.250 ± 0.214**
**Density**	1.008 ± 0.003	1.006 ± 0.004	1.0075 ± 0.003	1.004 ± 0.002

NC = normal control; DC = diabetic control; PE 500 = treated with *P. erinaceus* extract 500 mg/kg bw; Glib 0.6 = treated with glibenclamide 0.6 mg/kg bw. The urinary biochemical parameters were sought at the start and the end of the 41 days of treatment. Values were presented as mean ± SEM. n = 6.

Serum creatinine, AST and CPK were significantly (p < 0.01–0.0001) increased at the start of treatment in all diabetic rats of the different groups, compared to NC (Table 2). At the end of treatment, serum urea (p < 0.1), creatinine (p < 0.5), AST (p < 0.0001), ALT (p < 0.5) and the CPK (p < 0.0001) remained significantly elevated in the diabetic control compared to NC (Table 2). Compared to DC, the groups treated with the extract and the glibenclamide showed a significant reduction (p < 0.5–0.0001) of all these biochemical parameters except in ALT compared to DC (Table 2).

**Table 2:** Blood serum biochemical parameters.

Parameters	NC	DC	PE 500	Glib 0.6
**At the start of treatment**				
**Urea (g/L)**	0.505 ± 0.028	0.553 ± 0.012	0.589 ± 0.076	0.516 ± 0.063
**Cr (mg/L)**	6.268 ± 0.204	**9.304 ± 0.512**^**#**^	**9.800 ± 0.521**^**#**^	**8.565 ± 0.431**^**#**^
**ASAT (UI/L)**	54.120 ± 5.500	**165.967 ± 18.535**^**# #**^	**196.283 ± 16.469**^**#**^^**#**^^**#**^	**204.650 ± 19.506**^**#**^^**#**^^**#**^^**#**^
**ALAT (UI/L)**	60.087 ± 4.215	87.552 ± 9.412	65.592 ± 11.661	65.907 ± 11.642
**CPK (UI/L)**	389.145 ± 85.567	**910.700 ± 78,044**^**#**^^**#**^^**#**^^**#**^	**1013.000 ± 62.452**^**#**^^**#**^^**#**^^**#**^	**947.983 ± 67.146**^**#**^^**#**^^**#**^^**#**^
**At the end of the treatment**				
**Urea (g/L)**	0.586 ± 0.012	**1.180 ± 0.080**^**# #**^	0.823 ± 0.038*	0.640 ± 0.150***
**Cr (mg/L)**	7.035 ± 0.389	**9.576 ± 0.293**^**#**^	7.173 ± 0.300**	7.040 ± 0.710**
**ASAT (UI/L)**	119.297 ± 39.412	**367.362 ± 45.934**^**#**^^**#**^^**#**^^**#**^	245.000 ± 26.851***	211.000 ± 14.000***
**ALAT (UI/L)**	63.254 ± 5.362	**131.080 ± 14.709**^**#**^	125.000 ± 18.148	89.000 ± 4.000
**CPK (UI/L)**	489.886 ± 95.387	**1014.364 ± 72.915**^**#**^^**#**^^**#**^^**#**^	609.667 ± 33.198****	499.000 ± 78.000****

NC = normal control; DC = diabetic control; PE 500 = treated with *P. erinaceus* extract 500 mg/kg bw; Glib 0.6 = treated with glibenclamide 0.6 mg/kg bw. Cr = creatinine; AST = aspartate aminotransferase; ALT = alanine aminotransferase; CPK = creatine phosphokinase. Serum biochemical parameters were assessed at the start and end of 41 days of treatment. Values were analyzed with 1-way ANOVA and then presented as mean ± SEM. ^#^p < 0.05, ^# #^p < 0.01 vs. NC; *p < 0.05, **p < 0.01, ***p < 0.001 vs. DC. n = 6.

### 3.5 Hematological disorder

The complete blood count at the end of treatment showed a significant decrease (p < 0.001) in platelets and white blood cells in the diabetic control, compared to NC (Table 3). In the groups treated with the extract and glibenclamide, there was a significant increase in platelets (p < 0.01) and leukocytes (p < 0.001) compared to DC (Table 3).

**Table 3:** Hematological parameters.

Parameters	NC	DC	PE 500	Glib 0.6
**RBC (10**^**6**^**/μL)**	6.263 ± 0.384	6.543 ± 0.115	6.050 ± 0.179	6.035 ± 0.245
**HGB (g/dL)**	13.417 ± 0.910	13.567 ± 0.178	12.900 ± 0.598	13.050 ± 0.322
**HCT (%)**	35.517 ± 2.978	37.167 ± 0.924	34.550 ± 1.423	35.350 ± 1.583
**MCV (fl)**	56.333 ± 1.565	56.900 ± 0.755	57.065 ± 1.076	58.863 ± 0.967
**MCH (pg)**	21.367 ± 0.292	**20.757 ± 0.396**^**#**^	21.527 ± 0.475	22.500 ± 0.816
**MCHC (g/dL)**	38.083 ± 0.801	36.531 ± 0.789	37.488 ± 0.382	38.047 ± 1.842
**PLT (10**^**6**^**/μL)**	0.640 ± 0.036	**0.269 ± 0.041**^**# # #**^	0.510 ± 0,028**	0.587 ± 0.038**
**WBC (10**^**3**^**/μL)**	6.516 ± 0.180	**2.868 ± 0.160**^**# # #**^	5.383 ± 0.083***	5.550 ± 0.117***
**Neutrophils (%)**	42.167 ± 2.330	**30.833 ± 1.302**^**# #**^	37.000 ± 0.730	35.500 ± 2.110
**Eosinophils (%)**	3.167 ± 0.601	2.000 ± 0.516	2.667 ± 0.715	3.000 ± 0.365
**Basophils (%)**	0.000 ± 0.000	0.000 ± 0.000	0.000 ± 0.000	0.000 ± 0.000
**Lymphocytes (%)**	49.000 ± 3.246	**64.667 ± 1.498**^**# #**^	56.500 ± 1.455	57.833 ± 2.688
**Monocytes (%)**	6.000 ± 0.856	2.000 ± 0.577	3.833 ± 0.477	3.667 ± 0.615

NC = normal control; DC = diabetic control; PE 500 = treated with *P. erinaceus* extract 500 mg/kg bw; Glib 0.6 = treated with glibenclamide 0.6 mg/kg bw. RBC = red blood cells; HGB = hemoglobin level; HTC = hematocrit; MCV = mean corpuscular volume; MCHC = mean corpuscular hemoglobin content; MCHC = mean corpuscular hemoglobin concentration; PLT = platelets; WBC = white blood cells. A complete blood count was performed at the end of 41 days of treatment. Values were analyzed with 1-way ANOVA and then presented as mean ± SEM. # #p < 0.01, # # #p < 0.001 vs. NC; **p < 0.01, ***p < 0.001 vs. DC. n = 6.

### 3.6 Organ weight

**Fig. 5:**
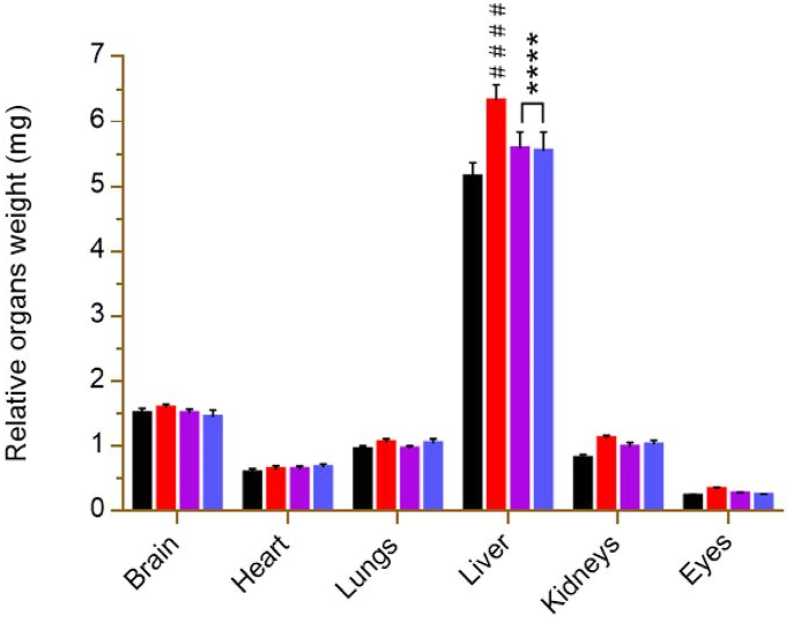
Effect of *P. erinaceus* on relative organ weights. NC = normal control; DC = diabetic control; PE 500 = treated with *P. erinaceus* extract 500 mg/kg bw; Glib 0.6 = treated with glibenclamide 0.6 mg/kg bw. Relative organ weights were measured on the 42nd day after the autopsy. Values were analyzed with 2-way ANOVA and then presented as mean ± SEM. ^# #^p < 0.01 vs. NC. n = 6.

At the end of the experiment, only the weight of the liver was significant (p < 0.001) in the diabetic control compared to the NC. However, the liver weights of the groups treated with the extract and glibenclamide did not show a significant decrease compared to DC (Fig. 5).

### 3.7 Histopathological studies

**Fig. 6:**
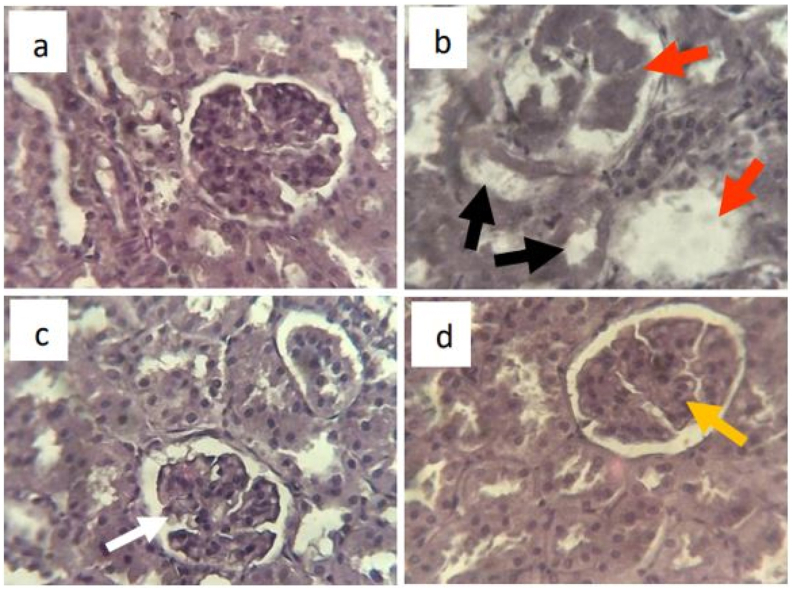
Histological sections of kidneys in rats at the end of treatment. a = normal control, shows normal architecture; b = diabetic control, shows diabetic nodular glomerulosclerosis (red arrow) and tubular fibrosis (black arrow); c = treated with *P. erinaceus* extract 500 mg/kg, shows normal glomerulus with enlarged Bowman's space (white arrow); d = treated with glibenclamide 0.6 mg/kg, shows enlarged glomeruli and Bowman's space (yellow arrow). Histological sections were performed on the kidneys fixed in 10% formalin after the autopsy on the 42nd day. H&E 400× staining under a light microscope. (For interpretation of the references to color in this figure legend, the reader is referred to the Web version of this article.)

Histological sections of kidneys in the normal control group showed normal architecture of glomeruli and tubules (Fig. 6a). In contrast, there was nodular glomerulosclerosis, progressive and complete loss of glomeruli and tubular fibrosis in the diabetic control group (Fig. 6b). In the group treated with the plant extract, a normal architecture of the glomeruli and the tubules was observed. Just an enlargement of Bowman's space could be observed (Fig. 6c). The glibenclamide-treated group has enlarged glomeruli and Bowman's spaces; the tubules have a normal architecture (Fig. 6d).

**Fig. 7:**
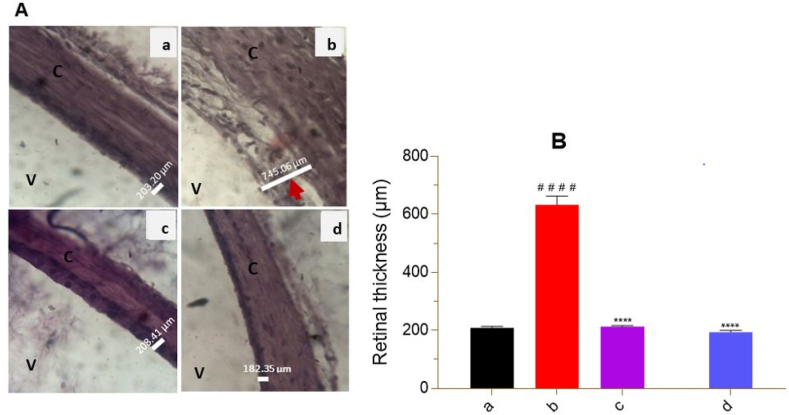
Histological sections and retinal thickness in rats at the end of treatment. A = histological section; B = retinal thickness. a = normal control; b = diabetic control; c = treated with *P. erinaceus* extract 500 mg/kg; d = treated with glibenclamide 0.6 mg/kg; V = vitreous body; C = choroid; White line = retinal thickness measurement; red arrow = macular edema. Histological sections were performed on the eyes fixed in 10% formalin after the autopsy on the 42nd day. H&E 400× staining under a light microscope. Values were analyzed with 1-way ANOVA and then presented as mean ± SEM. ^# # # #^p < 0.0001 vs. NC; ****p < 0.0001 vs. DC. n = 6. (For interpretation of the references to color in this figure legend, the reader is referred to the Web version of this article.)

Histological sections of the eyes showed well-demarcated normal retinas in the normal control group and the groups treated with the extract and glibenclamide. No detachment or alteration of the superficial retinal membrane was observed (Fig. 7A a, c and d). In contrast, in the diabetic control group, there was a sharp increase in retinal volume with detachment and alteration of the superficial membrane (Fig. 7A b). Measurement of retinal thickness in the diabetic control group showed a significant (p < 0.0001) increase of more than three and a half times (∼366.66%) compared to NC (Fig. 7B a). In the groups treated with the extract and glibenclamide, there was a significant (p < 0.0001) reduction in retinal thickness compared to DC (Fig. 7B c, d).

### 3.8 Oxidative stress

**Fig. 8:**
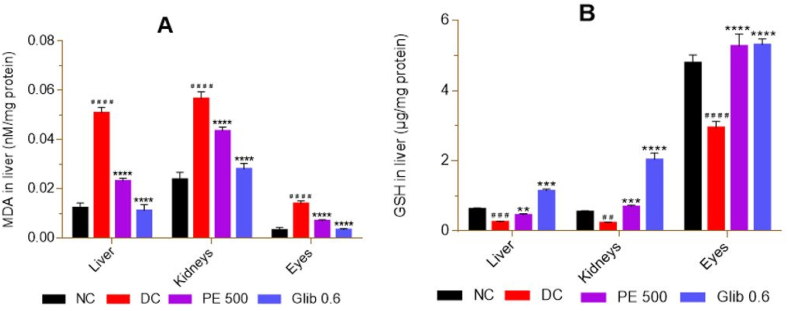
Effect of *P. erinaceus* extract on oxidative stress in rats. A = MDA; B = GSH. NC = normal control; DC = diabetic control; PE 500 = treated with *P. erinaceus* extract 500 mg/kg bw; Glib 0.6 = treated with glibenclamide 0.6 mg/kg bw. MDA and GSH were measured in the homogenates of the three organs after the autopsy on the 42nd day. Values were analyzed with 1-way ANOVA and then presented as mean ± SEM. ^# #^p < 0.01, ^# # #^p < 0.001, ^# # # #^p < 0.0001 vs. NC; **p < 0.01, ***p < 0.001, ****p < 0.0001 vs. DC. n = 6.

At the end of treatment, measurement of oxidative stress and antioxidant markers showed a significant increase (p < 0.0001) in MDA and GSH depletion in liver, kidney and eye homogenates, compared to NC (Fig. 8 A and B). When compared to DC, the groups treated with the extract and glibenclamide showed a significant decrease (p < 0.01–0.0001) in MDA and an increase in GSH levels in the liver, kidney and eye homogenates (Fig. 8 A and B).

## 4 Discussion

The antidiabetic property of the hydroethanolic extract of *P. erinaceus* stem bark was evaluated in a model of severe type 2 diabetes by the administration of streptozocin (STZ) to SD rats subjected to a hypercaloric diet (fructose + lard). The administration of fructose-lard for 20 consecutive days and the injection of streptozocin (on the 12th day) into the rats initially made it possible to obtain diabetic animals which complications. Curative treatment of diabetic rats with the plant extract for 3 consecutive weeks (from the 21st to the 41st day) subsequently made it possible to prove the effectiveness of *P. erinaceus* on type 2 diabetes and its complications.

Thus, the treatment of rats with fructose-lard for 20 consecutive days caused a metabolic syndrome which was revealed by a significant (p < 0.01–0.0001) increase in triglycerides, total cholesterol, LDL-C and TG/HDL-C ratio [22,23]. Significantly elevated triglyceride and cholesterol levels in obese subjects have been shown to promote insulin resistance and lead to diabetes [23,24]. Injecting streptozocin worsened insulin resistance in rats treated with fructose-lard and rapidly led to diabetes with blood glucose levels around 430 mg/dL. Indeed, streptozocin enters the pancreatic β-cell via the GLUT2 transporter and causes DNA alkylation leading to insulin resistance and the progressive destruction of pancreatic cells with the consequent onset of diabetes [[25], [26], [27]]. Maintaining the diabetic state in rats for approximately one week after injection of streptozocin resulted in complications that were revealed by urinary and serum biochemical parameters. Albimuniria (proteinuria) is a characteristic marker of nephropathy, as well as elevated serum urea and creatinine levels seen in renal failure [2,28]. The significantly elevated levels of the other urinary and serum biochemical parameters at the start of the treatment (on the 21st day) showed that the rats presented a severe diabetic state with cardiovascular complications.

Treatment of diabetic rats with *P. erinaceus* extract and glibenclamide improved insulin secretion and oral glucose tolerance, which was responsible for the significant (p < 0.0001) decrease in basal blood glucose in rats from the second week until the end of the treatment (from the 35th to the 42nd day). The oral glucose tolerance test performed at the end of the experiment on the 42nd day showed that the diabetic controls (DC) exhibited glucose intolerance, which was improved in rats treated with the plant extract and glibenclamide. This improvement in oral glucose tolerance corroborates the hypoglycemic activity of the plant previously obtained with the oral glucose tolerance test in ICR mice [13]. Diabetes is also characterized by excessive thirst, constant hunger and sudden weight loss [1,29]. These classic symptoms of diabetes were improved by treatment with plant extract and glibenclamide.

The complications induced by chronic hyperglycemia during diabetes are linked to the failure of several organs, such as the eyes, kidneys, liver, heart, lungs and brain [30]. Abnormalities of these organs can be observed macroscopically during an autopsy or by determining their weight. Measurement of relative organ weight after the autopsy on day 42 revealed no significant change (p > 0.05), thus suggesting protection of vital organs. This protective effect of *P. erinaceus* extract against diabetes complications was confirmed by histopathological, biochemical and hematological examinations.

Histological sections of the kidneys and eyes showed renal fibrosis and macular edema in the diabetic control group (DC). In rats treated with the plant extract at a dose of 500 mg/kg bw and glibenclamide at a dose of 0.6 mg/kg bw, no retinal abnormality was observed. In kidney sections, the widening of Bowman's space observed in rats treated with the plant extract is a minor and reversible anomaly that can be corrected in the long term [31]. It is thought that the glomerular hypertrophy seen in glibenclamide-treated rats may be related to the increased glomerular filtration rate (GFR) in this group. In diabetes, an elevated GFR occurs in the early stages with the changes in the glomerular filtration barrier which consists of glomerular endothelial cells, glomerular basement membrane and podocytes [9,32]. Gradually, there appears albuminuria, a decrease in GFR, and diffuse mesangial expansion, which progresses to lesions and nodular mesangial expansion; matrix protein deposition ultimately leads to glomerulosclerosis [9,33]. Tubulointerstitial fibrosis appears later and marks an irreversible stage that evolves toward ESRD [9,34]. Permanent and severe damage induced by hyperglycemia leads to renal fibrosis [9,35]. The protection observed in the treated groups would be due to the ability of the extract to reverse the process of renal fibrosis.

Diabetic retinopathy and nephropathy are strongly correlated and are marked by albuminuria [36,37]. In diabetes, the severity of retinopathy can predict the existence of nephropathy [37,38]. These two microvascular complications commonly encountered in diabetes gradually lead to blindness, renal failure and dialysis [1]. They invalidate the patient and compromise his vital prognosis [39]. Treatment with the plant extract was able to confer protection against nephropathy and retinopathy in this diabetic model. Thus, the extract of *P. erinaceus* at a dose of 500 mg/kg bw can prevent or reverse the progression to blindness and renal failure, which mark the end stages of diabetes complications.

In the kidneys, the disappearance at the end of the treatment of glycosuria, acetonuria, proteinuria, hematuria and leukocyturia on the one hand, and then the significant reduction (p < 0.05 and 0.01) of serum uremia and creatinine on the other hand, are in agreement with the absence of renal fibrosis observed on histological sections in treated groups with plant extract and glibenclamide. These biochemical parameters corroborate the protective effect of the plant extract against nephropathy in treated groups. The treatment also made it possible to avoid metabolic acidosis, which was revealed by the disappearance of acetonuria and the regulation of urinary pH toward normal in the groups treated with *P. erinaceus* extract and glibenclamide. It has been previously shown that in diabetes, a drop in pH below 7.20 due to the appearance and increase of ketone bodies in the blood and urine can lead to metabolic complications [40,41]. The presence of leukocyturia, significant leukopenia, a significant decrease in HDL-cholesterol and a significant increase in LDL-cholesterol in the diabetic control group (DC), are signs of inflammation that have been corrected in rats treated with the plant extract and glibenclamide. The hematuria results from high cystitis or a blood coagulation disorder which may be linked to the significant thrombocytopenia (p < 0.001) observed on the complete blood count at the end of treatment. These disorders were corrected by administrering of *P. erinaceus* extract and glibenclamide in diabetic rats.

In the eyes, macular edema can also be caused by the abnormal activation of metabolism induced by hyperglycemia, inflammation and lipoperoxidation. The significant increase in triglycerides and decrease in HDL-cholesterol in the diabetes control group (DC) are markers linked to retinal inflammation [42,43]. Administration of *P. erinaceus* extract and glibenclamide protected against retinal damage in diabetic rats. This protection involves the inhibition of pro-inflammatory precursors and oxidative stress.

The other biochemical parameters also revealed the protective effect of the plant extract against hepatic and cardiac damage. The significant increase in ALT and AST levels is an essential index of liver damage that may be caused by inflammation, as observed in the diabetic controls group (DC) [43]. The significant increase (p < 0.01) in triglyceride levels observed at the end of the experiment in the diabetic control group (DC) could lead to hepatic steatosis linked to the increase in liver volume and contribute to the worsening of insulin resistance [44]. This defective triglyceride metabolism impacted the serum cholesterol level, which resulted in a significant decrease (p < 0.05) in the HDL-C and a significant increase (p < 0.01) in the LDL-C which can reinforce insulin resistance and the risk of atherosclerosis [42,43]. Hepatic steatosis also increases oxidative stress through significant lipid metabolism [45]. Serum total CPK and proteinuria are predictive markers of heart and kidney damage [37,46]. Significantly elevated serum levels of CPK and LDL-cholesterol, as well as the presence of albuminuria, are often associated with cardiovascular events and nephrotic syndrome [46,47]. Administration of *P. erinaceus* extract and glibenclamide for three weeks caused a significant decrease (p < 0.001) in transaminases (ALT, AST), triglycerides, LDL-cholesterol and CKP in diabetic rats. Cell lipid deposition also leads to organ fibrosis [48,49]. Treatment with *P. erinaceus* extract can therefore prevent dyslipidemia, and hepatic steatosis and reduce the risk of cardiovascular disease.

In diabetes, hyperglycemia produces excessive oxygen free radicals (ROS), which induce oxidative stress and complications in several organs. Malondialdehyde (MDA) is a biomarker of lipoperoxydation, allowing the measurement of oxidative stress in a biological sample [50]. The process by which hyperglycemia causes retinopathy, nephropathy, and liver damage is similar and involves oxidative stress. Significantly (p < 0.001) elevated MDA levels and decrease in GSH in the eyes, kidneys and liver in the diabetic control group (DC) showed significant hyperglycemia-induced oxidative stress in these organs. In the present study, the effective (p < 0.001) reduction of MDA and increase of GSH in the homogenates of the eyes, kidneys and liver in treated diabetic rats proved the protective effect of *P. erinaceus* extract against complications related to oxidative stress. Other work on *P. erinaceus* has also shown that oxidative stress is one of the mechanisms leading to kidney damage [51]. Our previous *in vitro* studies revealed that the administration of the hydroethanolic extract of the stem bark of *P. erinaceus* at a dose of 500 mg/kg bw inhibited oxidative stress induced by high glucose levels [16].

In summary, the hydroethanolic extract of *P. erinaceus* stem bark at a dose of 500 mg/kg bw possesses a hypoglycemic activity comparable to that of glibenclamide and reverses the process of renal fibrosis and retinopathy in diabetes. These therapeutic properties of the plant could be attributed to phytochemicals such as polysaccharides and phenolic compounds (flavonoids, tannins) previously found in the extract [13]. Since the safety of the extract has been proven [52], the 500 mg/kg bw dose can then be used effectively to manage diabetes complications.

## 5 Conclusions

This study proved the efficacy of *P. erinaceus* extract against diabetes and its two microvascular complications, nephropathy and retinopathy, in a type 2 diabetes model induced by fructose-lard and streptozocin.

Data showed that the hypoglycemic activity of the plant extract was due to the significant reduction in basal blood glucose and improvement of oral glucose intolerance in diabetic rats. Biochemical and hematological parameters showed that treatment with the plant extract significantly reduced damage induced by hyperglycemia, particularly in the kidneys, eyes, liver and cardiovascular system. The protective effect of the plant extract on the kidneys and eyes has been confirmed by histological studies. Treatment with the extract conferred protection against renal fibrosis and macular edema. The decrease in blood glucose level, improvement in oral glucose intolerance, and inhibition of lipid deposition and fibrosis are the main mechanisms of action of *P. erinaceus* extract.

All the data proved the efficacy of the hydroethanolic extract of *P. erinaceus* stem bark on microvascular damage, mainly on diabetic nephropathy and retinopathy. However, molecular studies remain important to highlight the receptors on which the extract acts, then proceed to clinical trials and produce effective phytomedicines.

## Ethical approval of animal experiments

The experiments in rats were performed according to the guidelines for the use of research animals in the Laboratory of Animal Physiology at the University of Lome (001/2012/CB-MSDS-UL) in accordance with the internationally accepted principles for laboratory animal use and care, as set out in Directive 86/609/EEC.

## Author contributions

K.A. designed the methods, performed the work and wrote the manuscript. P.L-E. approved the methods and supervised the work. K.E-G. coordinated and approved the research. All authors read and approved the final manuscript.

## Funding

This research did not receive any specific grant from funding agencies in the public, commercial, or not-for-profit sectors.

## Declaration of competing interest

The authors declare that they have no known competing financial interests or personal relationships that could have appeared to influence the work reported in this paper.

## Data Availability

Data will be made available on request.
